# DCs facilitate B cell responses against microbial DNA via DC-SIGN

**DOI:** 10.1371/journal.pone.0185580

**Published:** 2017-10-04

**Authors:** Joris K. Sprokholt, Marieke H. Heineke, Tanja M. Kaptein, John L. van Hamme, Teunis B. H. Geijtenbeek

**Affiliations:** 1 Department of Experimental Immunology, Academic Medical Center, University of Amsterdam, Amsterdam, the Netherlands; 2 Amsterdam Infection & Immunity Institute, Amsterdam, the Netherlands; University of Bergen, NORWAY

## Abstract

Microbial DNA is highly immunostimulatory and is sensed by endosomal pattern recognition receptors after release from internalized microbes. It is unclear how extracellular DNA released from dead microbes is delivered to endosomal PRRs to induce immune responses. Here we have investigated the ability of DCs to bind and internalize extracellular *E*.*coli* DNA as well as synthetic DNA. DCs internalized *E*.*coli* and synthetic DNA, which was dependent on the C-type lectin receptor DC-SIGN. Notably, endosomal uptake of DNA by DCs enhanced TLR9-dependent responses of B cells against DNA. Hence, we have identified DC-SIGN as a cell surface receptor for DNA that facilitates immune responses directed against DNA.

## Introduction

Dendritic cells (DCs) are key players in sensing invading microbes and subsequent initiation of pathogen-specific adaptive immune responses. DCs sense conserved pathogen-associated molecular patterns (PAMPs) of microbes via pattern recognition receptors (PRRs), which induce innate signaling to activate DCs. DCs express numerous PRRs, including Toll-like receptors (TLRs) and C-type lectin receptors (CLRs) [1,2]. TLRs are either expressed as cell-surface receptors or as endosomal receptors and TLR localization is crucial for their activation and specificity [3,4]. TLR9 resides in endosomes and is activated by nonmethylated cytosine-guanine (CpG) motifs, which are twenty times more abundant in microbial DNA compared to mammalian DNA [5]. However, microbial DNA is only accessible to TLR9 after degradation of microbes in endolysosomal compartments, which adds to the specificity of TLR9 and prevents activation by CpG motifs within self DNA as self DNA is normally not present in endolysosomal vesicles [6].

Depending on the ligand, TLR9 can induce type I interferon (IFN) responses or cytokines responses that are critical in immunity against viruses and bacteria [7,8]. Synthetic CpG oligonucleotides (ODN) resemble microbial DNA and activate TLR9. The immunostimulatory properties of CpG ODN depend on the number of CpG motifs, nucleotide sequence, presence of poly-G sequence, single or double-stranded nature and the presence of a phosphorothioate backbone [5]. CpG ODNs are divided into different classes based on these characteristic. Class A ODN contain a poly-G sequence which leads to spontaneous formation of large aggregates that are retained longer in early endosomes and hence induce high levels of type I IFN but low levels of NFκB activation. Class B ODNs are linear structures and contain a phosphorothioate backbone and induce strong NFκB activation and moderate type I IFN induction. Class C ODNs also have a phosphorothioate backbone, form duplex structures and induce intermediate responses compared to class A and B ODN [8,9].

NFκB activation by CpG ODN leads to strong cytokine responses and maturation of myeloid DCs. Consequently, class B ODN are extensively studied as vaccine adjuvant and are currently in phase I/II clinical trials [10]. Although class A and C ODN are not preferred over class B ODN as vaccine adjuvant, they have been used to prevent or treat a number of diseases. Class C ODN has been used as monotherapy for hepatitis C virus (HCV)-infected individuals and administration lowered viral levels in blood [11]. Moreover, rhesus macaques were protected against *Leishmania amazonensis* infections when class A ODN was administered before and during the course of infection, while class B ODN did not protect against *Leishmania amazonensis* infections [12].

Despite that extracellular microbial DNA is highly immunostimulatory, it is unclear how extracellular microbial DNA is internalized to activate TLR9. For synthetic DNA it was recently shown that CLR DEC-205 and mannose receptor (MR) function as uptake receptors for CpG ODNs in mice [13,14]. DEC-205 binding is limited to class B and C ODNs but does not bind class A ODNs, while MR recognizes all classes of ODN [13,14]. However, less is known about the function of DEC-205 and MR in CpG ODN uptake in humans and it is unclear if these receptors are able to internalize extracellular microbial DNA. Human DNA can be opsonized by the antimicrobial peptide LL37, which protects self-DNA from degradation by extracellular nucleases and results in endocytosis and type I IFN responses via plasmacytoid DCs [15,16]. However, LL37 is produced by keratinocytes and neutrophils in the skin and it is unclear if LL37 is involved in the uptake of DNA in other tissues or by other DC subsets than plasmacytoid DCs.

CLR DC-SIGN contains an internalization motif and DC-SIGN-ligand complexes are targeted to endolysosomal compartments resulting in antigen presentation to T cells [17]. DC-SIGN is expressed on DCs and macrophages and is involved in numerous immune processes, including pathogen uptake, innate signaling and shaping adaptive immune responses [1,2,18] DC-SIGN has broad ligand-specificity and recognizes mannose, fucose and GlcNAc structures, which commonly occur as repetitive structures on glycosylated proteins or lipids. As DNA is a repetitive structure we investigated whether DC-SIGN is able to bind extracellular DNA.

Here we show that DC-SIGN binds *E*. *coli* DNA directly in a Ca^2+^-dependent manner and that DCs require DC-SIGN to internalize *E*. *coli* DNA. Notably, DC-SIGN facilitated microbial DNA-induced cytokine responses. We recapitulated these findings with synthetic DNA and show that DC-SIGN plays a central role in the ability of DCs to bind and internalize class A ODNs. Moreover, DCs enhanced B cell responses against DNA via DC-SIGN. Hence, we have identified DC-SIGN as a key binding receptor for synthetic and microbial DNA in human DCs.

## Results

### DC-SIGN binds synthetic ODN and microbial DNA

**Fig 1:**
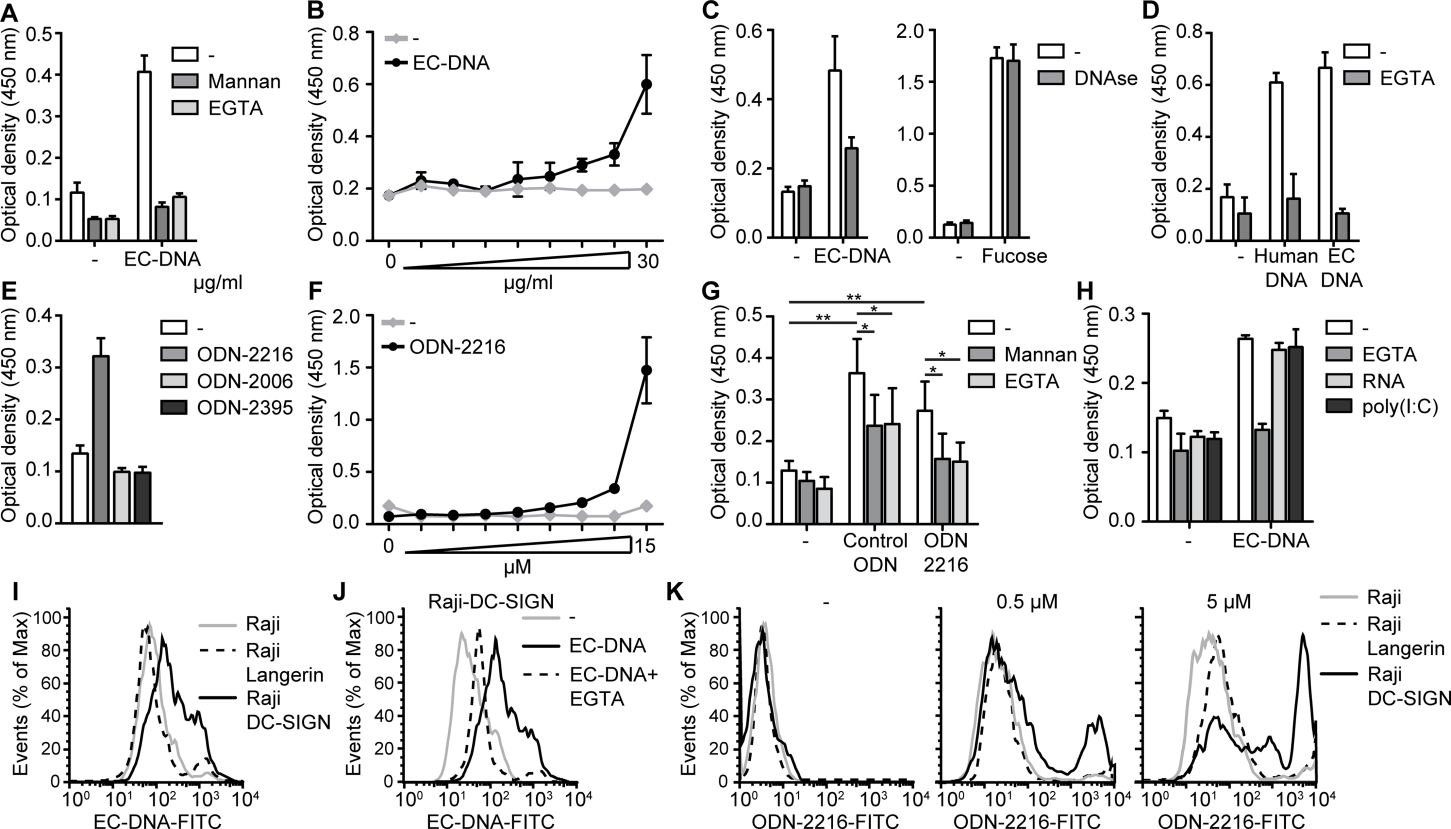
DC-SIGN binds class A ODN and microbial DNA. (**A**,**B**,**D**-**H**) *E*. *coli* DNA (**A**,**B**,**D**), human DNA (**D**) or indicated ODNs (**E-H**) were coated on high binding plates and recombinant DC-SIGN binding to coated ligands was measured by ELISA. (**C**) Recombinant DC-SIGN was coated on high binding plates and binding to DNAse-treated or untreated biotin-labeled *E*. *coli* DNA or Fucose was measured by ELISA. (**I-K**) Binding of parental Raji cells or Raji cells stably expressing DC-SIGN or Langerin to FITC-labeled *E*. *coli* DNA (**I,J**) or FITC-labeled ODN-2216 (**K**) was analyzed by flow cytometry. 10 μg/ml DNA or 5 μM ODN was used in all experiments unless stated otherwise. Data are collated (mean ± s.d.) of four independent experiments (**G**) or representative of at least four (**I**), three (**E**) or two (**A-D,F,H**,**J**,**K**) independent experiments (mean ± s.d. of duplicates in **A**-**F,H**). *P<0.05, **P<0.01 (student’s t-test). EC-DNA: *E*. *coli* DNA.

We and others have shown that DC-SIGN by recognizing mannose and fucose structures, binds a diverse range of proteins and carbohydrate structures [2,19–21]. To investigate whether DC-SIGN binds DNA structures, *E*. *coli* DNA was immobilized on high-binding plates and interaction with recombinant DC-SIGN was measured. Strikingly, DC-SIGN bound microbial DNA, which was blocked by mannan, a competitive inhibitor of DC-SIGN binding, confirming the specificity of the interaction (Fig 1A and 1B). DC-SIGN binding to carbohydrate structures is Ca^2+^-dependent [22]; and the interaction between DC-SIGN and microbial DNA was also Ca^2+^-dependent as EGTA abrogated binding (Fig 1A). To exclude that DC-SIGN binds to any impurities we used microbial DNA from a commercial source that guarantees endotoxin levels below 0.001ng/μg DNA, which we confirmed using TLR2 or TLR4-transfected HEK cells (S1 Fig). To further confirm that DC-SIGN-DNA binding is specific, we treated *E*. *coli* DNA with DNAse, which abrogated DC-SIGN binding to microbial DNA while DNAse did not affect DC-SIGN binding to DC-SIGN ligand fucose (Fig 1C). Notably, DC-SIGN bound human DNA to a similar extent as *E*. *coli* DNA, indicating that CpG motifs are not involved in binding (Fig 1D).

Microbial DNA contains many CpG domains that are resembled by synthetic CpG ODNs. Therefore we investigated whether CpG ODN is recognized by DC-SIGN. Different CpG classes, class A, B and C ODN, were coated on high-binding plates and DC-SIGN binding was measured. Interestingly, recombinant DC-SIGN bound class A ODN-2216 in contrast to class B or C ODN-2006 and ODN-2935, respectively (Fig 1E and 1F). The binding of DC-SIGN to class A ODN was blocked by both mannan and EGTA (Fig 1G). Next, we investigated the importance of CpG-motifs in DC-SIGN-ODN binding using control ODN that is similar in length and structure but lacks CpG-motifs. DC-SIGN was able to bind control ODN in a Ca^2+^-dependent manner and this was inhibited by mannan, indicating that DC-SIGN binds ODN in a CpG-independent manner (Fig 1G).

DNA and synthetic ODNs are predominantly negatively charged due to the phosphodiester backbone. To investigate if a negative charge is sufficient for DC-SIGN binding we used RNA and the synthetic equivalent poly(I:C) to block DNA binding. However, pre-incubating DC-SIGN with RNA or poly(I:C) did not affect the binding to *E*. *coli* DNA, indicating that a negative charge is not sufficient for DC-SIGN binding (Fig 1H).

To examine whether DC-SIGN was also important for cellular binding of microbial DNA we used Raji cells which stably expressed DC-SIGN or CLR Langerin on the cell surface and measured binding to FITC-labeled microbial DNA by flow cytometry. Strikingly, Raji-DC-SIGN cells bound microbial DNA in contrast to parental Raji cells or Raji-Langerin cells (Fig 1I). Moreover, EGTA strongly decreased microbial DNA binding to Raji-DC-SIGN cells (Fig 1J). DC-SIGN was also required for cellular binding of class A ODN as Raji-DC-SIGN in contrast to Raji and Raji-Langerin interacted with class A ODN (Fig 1K). Together these data strongly indicate that DC-SIGN is a receptor for synthetic and biological DNA independent of methylation status.

### DC-SIGN facilitates DNA uptake by dendritic cells

**Fig 2:**
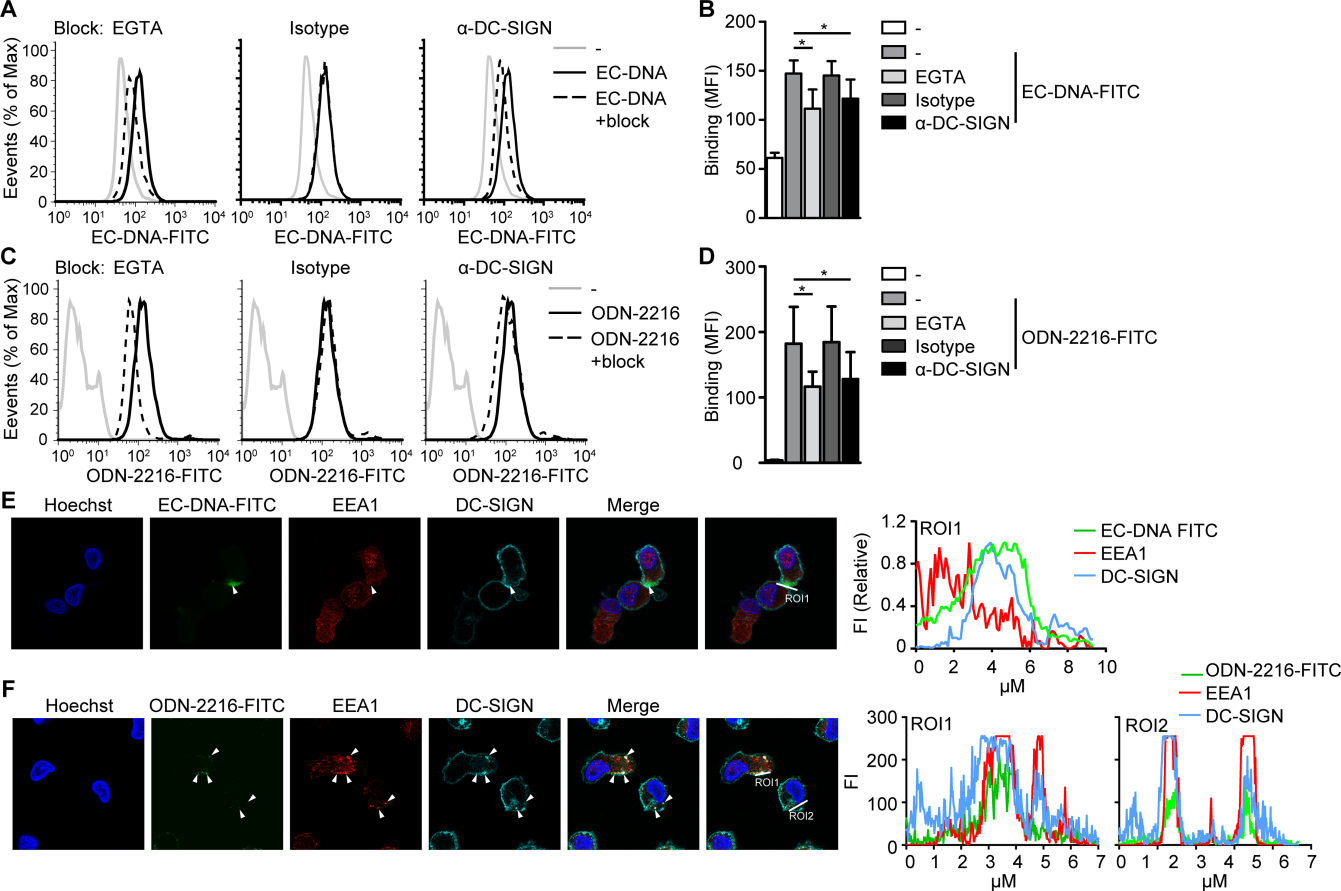
Dendritic cells interact with both class A ODN and microbial DNA via DC-SIGN. (**A**-**D**). Flow cytometry analysis of monocyte-derived DCs incubated with EC-DNA-FITC (**A,B**) or ODN-2216-FITC (**C,D**) for 10 min in the presences or absence of EGTA, IgG1 isotype control or blocking antibodies directed against DC-SIGN. (**E**,**F**) Confocal imaging of EC-DNA-FITC (green, **E**) or ODN-2216-FITC (green, **F**), early endosome antigen 1 (EEA1, red), DC-SIGN (turquoise) and DNA (Hoechst, blue) in monocyte-derived DCs stimulated with EC-DNA-FITC (**E**) or ODN-2216-FITC (**F**). 10 μg/ml DNA or 5 μM ODN was used in all experiments. Data are collated (mean ± s.d.) of three (**B**,**D**) independent experiments with different donors or are representative of at least three (**A,C**) or two (**E,F**) independent experiments with different donors. *P<0.05, **P<0.01 (student’s t-test).EC-DNA: *E*.*coli* DNA, ROI: region of interest.

Extracellular DNA can activate DCs [23].Therefore we investigated whether human monocyte-derived DCs that express high levels of DC-SIGN interact with class A ODN and microbial DNA. DCs interacted with class A ODN as well as microbial DNA, which was partly dependent on Ca^2+^ (Fig 2A–2D). Moreover, blocking antibodies directed against DC-SIGN decreased binding of class A ODN and microbial DNA by DCs (Fig 2A–2D). Next, we followed internalization of DNA ligands into endosomal compartments where they can trigger endosomal TLRs [3]. DC-bound microbial DNA colocalized with DC-SIGN, but we were unable to detect internalized DNA (Fig 2E). Most likely because of low fluorescence of FITC-labeled DNA and rapid degradation after endosomal uptake. Therefore, we switched to FITC-labeled class A ODN, which has high stability and fluorescence. DCs internalized class A ODN and this co-localized with early endosome antigen 1 (EEA1) and DC-SIGN, indicating that class A ODN is delivered to DC-SIGN^+^ endosomes (Fig 2F). These data show that DC-SIGN facilitates binding of synthetic and microbial DNA by DCs and that binding leads to endosomal delivery of DNA.

### Synthetic and microbial DNA induce type I IFN and cytokine responses

**Fig 3:**
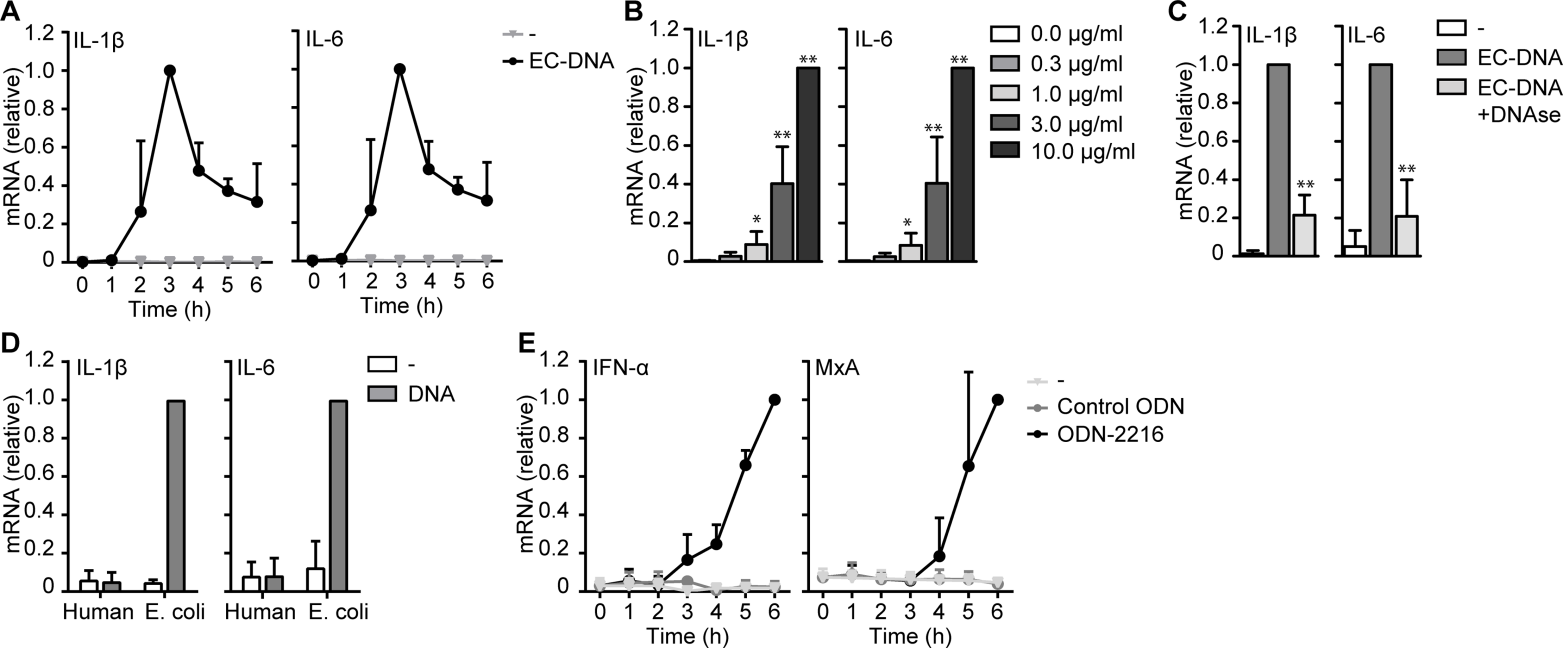
Dendritic cells produce type I IFN or cytokines in response to synthetic and microbial DNA. (**A**,**B**,**D**,**E**) mRNA analysis of monocyte-derived DCs stimulated with EC-DNA (**A**,**B**,**D**), human DNA (**D**), ODN-2216 or control ODN (**E**) for indicated time points was measured by real-time PCR, normalized to GAPDH and set as 1 in samples with the highest expression. (**C**) Similar as in (**A**), but EC-DNA was treated with DNAse before stimulation. Cells were stimulated with 10 μg/ml DNA or 5μM ODN in all experiments unless stated otherwise. Data are collated (mean ± s.d.) of four (**C**), three (**A**,**B**) or two (**D**,**E**) independent experiments with different donors *P<0.05, **P<0.01 (student’s t-test). EC-DNA: *E*. *coli* DNA.

CpG ODN and microbial DNA are known to induce type I IFN and/or cytokine responses, depending on the class of CpG ODN used [5]. We examined microbial DNA-induced gene expression over time in DCs. *E*. *coli* DNA induced IL-1β and IL-6 expression which peaked around 3 hours post stimulation (Fig 3A). Titrating *E*. *coli* DNA to determine the sensitivity indicated that DCs respond to concentrations of *E*. *coli* DNA as low as 1 μg/ml (Fig 2B). Moreover, *E*. *coli* DNA-induced IL-1β and IL-6 responses were abrogated by DNAse treatment, strongly suggesting that DNA itself and not contaminants triggered these responses (Fig 3C). *E*. *coli* DNA-induced responses most likely also involved CpG motifs as human DNA did not induce IL-1β or IL-6 expression in DCs (Fig 3D). In contrast to microbial DNA, class A ODN induced IFN-α and we could not detect IL-1β or IL-6 induction (Fig 3E). IFN-α secretion leads to IFNα/β receptor (IFNα/βR) signaling and the induction of interferon stimulated genes (ISGs) [24]. Class A ODN induced expression of ISG MxA indicating that class A ODN triggers functional type I IFN responses. Consistent with our observations with human DNA, control ODN, lacking CpG motifs, did not induce type I IFN responses (Fig 3E). These data strongly indicate that microbial DNA and class A ODN induce distinct immune responses in DCs upon internalization and that these effects involve CpG motifs.

### DC-SIGN facilitates synthetic and microbial DNA induced responses

**Fig 4:**
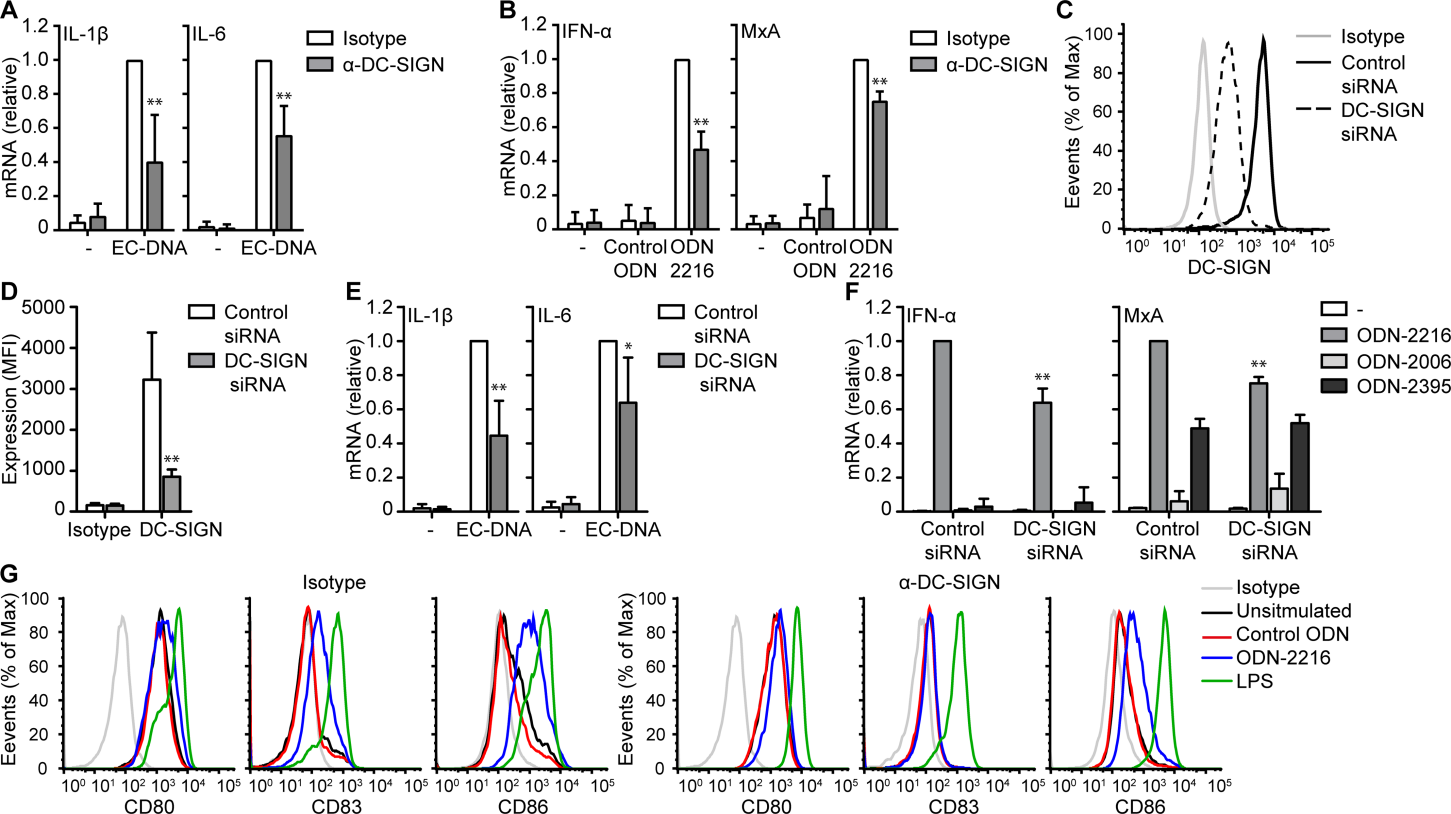
DC-SIGN facilitates microbial DNA induced responses. (**A**,**B,E,F**) mRNA analysis of monocyte-derived DCs stimulated with EC-DNA (**A,E**), ODN-2216 or control ODN (**B,F**) in the presence or absence of IgG1 isotype control or blocking antibodies against DC-SIGN (**A**,**B**) or after treatment with control or DC-SIGN siRNA (**E,F**) was measured by real-time PCR, normalized to GAPDH and set as 1 in samples with IgG1 isotype control or control siRNA. (**C**,**D**) DC-SIGN expression of monocyte-derived DCs after control or DC-SIGN siRNA treatment was measured by flow cytometry. (**G**) Expression of CD80, CD83 and CD86 expression by monocyte-derived DCs stimulated with control ODN or ODN-2216 in the presence or absence of IgG1 isotype control or blocking antibodies against DC-SIGN. Cells were stimulated with 10 μg/ml DNA or 5μM ODN in all experiments. Data are collated (mean ± s.d.) of six (**D**), four (**A,B,**) or three (**E,F**) independent experiments with different donors or are representative of six (**C**) or two (**G**) independent experiments with different donors. *P<0.05, **P<0.01 (student’s t-test). EC-DNA: *E*. *coli* DNA.

Next, we investigated DC-SIGN function in microbial DNA-induced cytokine responses using neutralizing antibodies against DC-SIGN. Strikingly, inhibiting DC-SIGN reduced the induction of IL-1β and IL-6 by *E*. *coli* DNA (Fig 4A). Also class A ODN-induced responses involved DC-SIGN as neutralizing DC-SIGN decreased IFN-α and MxA expression (Fig 4B). Neutralizing antibodies against DC-SIGN significantly reduced but did not abrogate microbial DNA and class A ODN-induced responses, possibly because of the involvement of different or multiple binding sites in DC-SIGN that are not blocked by the antibody. Therefore we used RNA interference to silence DC-SIGN expression (Fig 4C and 4D). Silencing DC-SIGN significantly reduced microbial DNA and class A ODN induced immune responses to a similar extent as neutralizing antibodies (Fig 4E and 4F). Interestingly, DC-SIGN silencing only reduced type I IFN responses induced by class A ODN and not by class C ODN, which does not bind to DC-SIGN (Fig 4F). Class C ODN induced less IFN-α and MxA than class A ODN, consistent with previous studies were class A ODNs induce strong type I IFN responses while class C ODN induce moderate type I IFN responses ([8,9].

DC maturation is a key process for the induction of adaptive immune responses and we examined the expression of maturation molecules CD80, CD83 and CD86 after class A ODN stimulation. Class A ODN induced increased expression of CD83 and CD86 (Fig 4G). Notably, neutralizing antibodies against DC-SIGN reduced ODN-2216-induced CD83 and CD86 expression (Fig 4G). These data strongly indicate that DC-SIGN facilitates microbial DNA and class A ODN induced responses.

### DC-SIGN facilitates DNA induced responses by B cells

**Fig 5:**
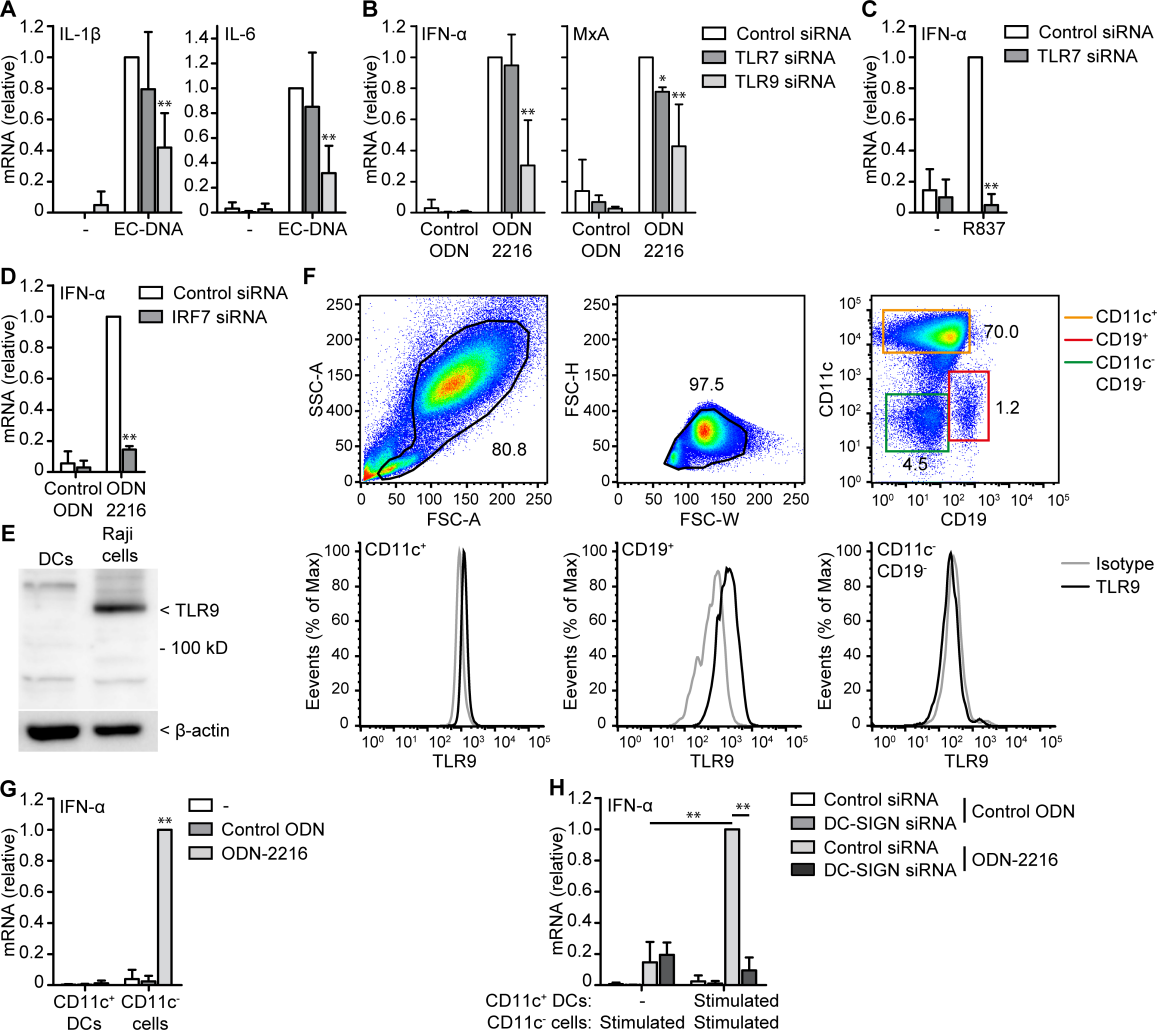
CD11c^+^ DCs enhance IFN-α production of CD11c^-^ cells via DC-SIGN. (**A-D**) mRNA expression of monocyte-derived DCs stimulated with EC-DNA (**A**), control ODN, ODN-2216 (**B,D**), or R837 (**C**) after treatment with control, TLR7, TLR9 (**A-C**), or IRF7 siRNA (**D**) was measured by real-time PCR, normalized to GAPDH and set as 1 in samples with control siRNA. (**E**) Immunoblot of monocyte-derived DCs or Raji cells whole cell lysate for TLR9. β-actin was used as loading control. (**F**) Analysis of TLR9 expression in monocyte-derived DC culture by flow cytometry. Number adjacent to gates indicates percentage of gated cells. (**G**) mRNA expression of sorted CD11c^+^ DCs and CD11c^-^ cells stimulated with control ODN or ODN-2216 was measured by real-time PCR, normalized to GAPDH and set as 1 in samples with ODN-2216 of CD11c^-^ cells. (**H**) mRNA expression of sorted CD11c^+^ DCs and CD11c^-^ cells stimulated with control ODN or ODN-2216 after treatment with control or DC-SIGN siRNA. Sorted cells were stimulated for 1h, washed and combined for 5h before mRNA expression was measured by real-time PCR, normalized to GAPDH and set as 1 in samples with control siRNA. Cells were stimulated with 10 μg/ml DNA or 5μM ODN in all experiments. Data are collated (mean ± s.d.) of four (**A,B,G**), three (**H**) or two (**C,D**) independent experiments with different donors or are representative of four (**F**) or two (**E**) independent experiments with different donors. *P<0.05, **P<0.01 (student’s t-test). EC-DNA: *E*. *coli* DNA.

Microbial DNA and synthetic DNA induce immune responses via TLR9 in different human immune cells including specific dendritic cell subsets and B cells [5]. We used RNA interference to silence TLR9 to investigate if microbial DNA and class A ODN-induced responses are TLR9-dependent (S3 Fig). Silencing of TLR9 significantly reduced microbial DNA-induced IL-1β and IL-6 as well as decreased class A ODN induced IFN-α and MxA expression (Fig 5A and 5B). Silencing of TLR7 only minimally affected MxA induction after class A ODN stimulation (Fig 5A and 5B), while IFN-α induction by TLR7 ligand R837 was abrogated (Fig 5C). TLR9 induces type I IFN responses via transcription factor IRF7 and silencing of IRF7 abrogated class A ODN-induced IFN-α expression (Fig 5D). These data strongly indicate that synthetic and microbial DNA-induced responses are mediated by TLR9. However, we were unable to detect TLR9 expression in monocyte-derived DCs by immunoblot while we detected TLR9 expression in Raji cells, which are immortal B cells and express high levels of TLR9 (Fig 5E).

Monocyte-derived DC cultures can contain small percentages of other cells and therefore we investigated TLR9 expression using flow cytometry. This revealed that a small percentage of CD19^+^ B cells (1,54% ± 0.46) was present in the monocyte-derived DC culture and that only CD19^+^ B cells, but not CD11c^+^ DCs nor other cells (CD19^-^CD11c^-^) expressed TLR9 (Fig 5F). Next, we sorted CD11c^+^ DCs and CD11c^-^ cells containing B cells and stimulated both fractions with class A ODN. Notably, ODN-2216 induced IFN-α in CD11c^-^ cells and not in CD11c^+^ DCs (Fig 5G). These results indicate that TLR9^+^ B cells and not CD11c^+^ DCs induce type I IFN responses against class A ODN in the monocyte-derived DC culture. This was surprising because only CD11c^+^ DCs express DC-SIGN (S4 Fig) while neutralizing antibodies or RNA interference directed against DC-SIGN significantly decreased ODN-2216 induced IFN-α (Fig 4E and 4F). Altogether, these results suggest that not only DC-SIGN-mediated uptake of DNA in CD11^+^ DCs, but also TLR9-mediated sensing in B cells is required for DNA sensing in monocyte-derived DC cultures.

It is known that DCs can stimulate B cell responses and transfer antigen to B cells and our findings further uncover an important role for DC-B cell crosstalk in type I IFN production upon DNA sensing [25,26]. To investigate if DCs stimulate B cells in ODN-2216-induced responses via DC-SIGN we sorted CD11c^+^ DCs and CD11c^-^ cells and pulsed CD11c^-^ cells with ODN-2216. Co-culture of untreated CD11c^+^ DCs with ODN-stimulated CD11c^-^ cells resulted in IFN-α induction and this IFN-α induction was independent of DC-SIGN. By contrast, when ODN-stimulated CD11c^+^ DCs were combined with ODN-stimulated CD11c^-^ cells we observed a stronger increase in IFN-α expression compared to CD11c^-^ pulsed alone and this IFN-α augmented response was dependent on DC-SIGN (Fig 5H). These data suggest that pulsed CD11c^+^ DCs enhance the type I IFN response of CD11c^-^ cells against class A ODNs and that this enhancement is dependent on DC-SIGN.

## Discussion

Microbial DNA is a potent inducer of immune responses and TLR9-mediated recognition of microbial DNA is pivotal for effective immunity against pathogenic microbes [27–29]. However, it is unclear how TLR9-mediated responses against extracellular microbial DNA are facilitated by cell surface receptors. Here we identified DC-SIGN as a cell surface receptor for microbial and human DNA. DC-SIGN-mediated uptake resulted in endosomal delivery of DNA and induction of type I IFN and cytokine responses in response to microbial DNA. Moreover, DC uptake of synthetic DNA, which resembles microbial DNA, also involved DC-SIGN. TLR9 expression was confined to a small fraction of CD19^+^ B cells in the DC culture and these cells were responsible for type I IFN responses induced by synthetic DNA. Interestingly, DCs enhanced synthetic DNA-induced responses of B cells via DC-SIGN. These data indicate that DC-SIGN facilitates DNA recognition by DCs and that DCs enhance DNA-induced immune responses of B cells via DC-SIGN.

DEC-205 has been identified as an uptake receptor for ODN in mice that facilitates DC maturation and B cell activation in response to synthetic ODNs and recombinant human DEC-205 was shown to interact with synthetic ODNs [13]. However, human and mice DEC-205 only bound class B and C ODN and not class A ODN. Our data shows that DC-SIGN exclusively binds class A ODN, which suggests that DC-SIGN and DEC-205 could function as complementary receptors for synthetic ODNs in humans. Class A ODN differ from class B and C ODN in containing a poly-G sequence that results in spontaneous formation of large aggregates with repetitive structures. The avidity of DC-SIGN for repetitive structures by forming tetramers at the cell surface might explain the binding affinity for class A ODN and microbial DNA over class B or C ODNs [30]. In addition, the negative charge of DNA could be involved in DC-SIGN-DNA binding although our data show that DC-SIGN does not bind to RNA, which also has a negative charge. This indicates that a negative charge is not sufficient for DC-SIGN binding.

The binding sites of DC-SIGN for carbohydrate structures have been well-defined [31]. Our data show that similar to carbohydrate structures, the binding of DNA by DC-SIGN is dependent on calcium. However, it is unclear if DC-SIGN utilizes the same binding sites for DNA as for carbohydrates. Moreover, the binding sites used for biological or synthetic DNA might differ as the poly-G sequence and phosphorothioate backbone of synthetic DNA are not present in biological DNA.

Class A ODNs are characterized as strong inducers of type I IFN because they are retained for longer periods in early endosomes compared to class B and C ODN [8,9]. Indeed, we detected high levels of IFN-α in DCs in response to class A ODN stimulation and no detectable levels of IL-1β and IL-6 expression. Interestingly, microbial DNA did not induce type I IFN responses indicating that it is rapidly shuttled to lysosomes. Rapid processing of nucleic acids leads specifically to NFκB but not IRF7 activation, which could explain the lack of type I IFN responses [9].

Our data show that the responses against microbial DNA and class A ODN critically depended on CpG motifs, as human DNA or control ODN lacking CpG motifs did not induce type I IFN or cytokine responses. The importance of CpG motifs is consistent with the ligand specificity of TLR9 [27]. Indeed, silencing TLR9 strongly reduced microbial and synthetic DNA-induced responses, indicating that the responses are induced by CpG-containing DNA and not by possible contaminants. Moreover, our data show that these responses are facilitated by DC-SIGN as neutralizing antibodies and RNA interference directed against DC-SIGN significantly reduced the responses against microbial and synthetic DNA. Interestingly, in depth analysis of the monocyte-derived DC culture indicated that TLR9 expression was confined to a small fraction of CD19^+^ B cells while DC-SIGN was exclusively expressed by CD11c^+^ DCs.

This could indicate that DCs facilitate B cell responses via DC-SIGN intrinsically or that DCs require DNA stimulation to enhance B cell responses against DNA via DC-SIGN. Our data indicate the latter as only synthetic DNA-stimulated DCs enhance B cell responses and this was depended on DC-SIGN.

Our data indicate that DCs facilitate B cells for optimal responses against synthetic DNA via DC-SIGN. Although we did not investigate the requirement of cell-cell contact for DC-enhanced B cell responses, antigen transfer from DCs to B cells has been described before and could be involved [25,26]. In addition, it has been shown that B cells poorly internalize and respond to class A ODN. However, in complex with antibodies class A ODN is efficiently internalized and induces similar B cell responses as class B ODN, indicating that B cells respond suboptimal to class A ODN without additional signals [32]. Our data suggest that DCs could provide those signals via DC-SIGN either by transferring antigen to B cells or unknown activation signals. How B cells internalize DNA in the absence of DCs remains unclear.

DC-SIGN binding to synthetic and microbial DNA was independent of CpG motifs and we observed that DC-SIGN also strongly bound to human DNA. However, human DNA did not induce type I IFN or cytokine responses in DCs. LL37 opsonization of human DNA leads to robust type I IFN responses by plasmacytoid DCs via TLR9, because DNA-LL37 complexes form condensed aggregates that are retained in early endosomes [15]. This could indicate that DC-SIGN binding to human DNA does not lead to aggregation and therefore does not trigger TLR9. However, reactive oxygen species-induced oxidative damage of extracellular DNA increases its resistance to nucleases and injection of oxidative DNA into skin leads to immune activation [33]. This process could involve DC-SIGN-mediated uptake of oxidative DNA by DCs as our data show that DC-SIGN facilitates B cells responses against synthetic DNA. As extracellular DNA plays a central role in several autoimmune diseases, including lupus and psoriasis, our data does not only have implications for pathogenic diseases but for autoimmune diseases as well. Targeting DC-SIGN-DNA binding could be an effective strategy to improve patient care of patients suffering from lupus or psoriasis.

Our data shows that neutralizing DC-SIGN significantly lowered responses to and binding of class A ODN and microbial DNA by monocyte-derived DCs, but was unable to abrogate these responses, suggesting that other pathways or receptors might be involved in internalization. Non-specific endocytosis or pinocytosis has been proposed to contribute to CpG ODN internalization *in vitro*, although this does not appear to play a role *in vivo* [13]. Pinocytosis could also be responsible for the small fraction of Raji and Raji-Langerin cells that bound microbial DNA and class A ODN. In particular because a similar fraction of DNA-binding cells remained after successful blocking of microbial DNA-binding by Raji-DC-SIGN cells using EGTA.

DC-SIGN recognizes several bacteria via carbohydrate structures present on the outside of bacteria [19,34,35]. We have now identified that microbial DNA is an additional ligand for DC-SIGN and thereby greatly expand the potential function of DC-SIGN in numerous diseases. Extracellular microbial DNA occurs when bacteria are lysed (e.g. complement activation) or when bacteria form biofilms. These structures are notoriously resistant against immune clearing and underlie the majority of persistent bacterial infections in humans [36]. Extracellular bacterial DNA plays a central role in the initiation of biofilms by facilitating bacterial adhesion and in the formation of bacterial aggregates after adhesion [37,38]. Targeting the immune system to bacterial DNA and DNA-associated proteins has been shown to be successful in dispersing biofilms [39,40]. DC-SIGN could provide DCs with the capacity to bind and internalize extracellular bacterial DNA and associated protein and possibly aid in biofilm clearance, by producing inflammatory mediators and presenting antigen of DNA-associated proteins for effective adaptive immune responses.

In summary, we have identified DC-SIGN as a cell surface receptor for microbial DNA, which could be important in sensing microbial lysis and biofilms. Moreover, DCs enhanced B cell responses against DNA via DC-SIGN and targeting of DC-SIGN has the potential to improve vaccination strategies.

## Material and methods

### Ethics statement

This study was done in accordance with the ethical guidelines of the Academic Medical Center and human material was obtained in accordance with the AMC Medical Ethics Review Committee (i.e. Institutional Review Committee) according to the Medical Research Involving Human Subjects Act. Buffy coats obtained after blood donation (Sanquin) is not subjected to informed consent according to the Medical Research Involving Human Subjects Act and the AMC Medical Ethics Review Committee. All samples were handled anonymously.

### Cell isolation and stimulation

Peripheral blood monocytes were isolated from buffy coats of healthy donors (Sanquin) by Lymphoprep (Axis-Shield) gradient followed by Percoll (Amersham Biosciences) gradient isolation. Monocytes were differentiated into immature DCs in by the addition of 800 U/ml GM-SCF and 500 U/ml IL-4 (both Invitrogen) for 6–7 days in RPMI supplemented with 10% fetal calf serum, 10 U/ml penicillin, 10 mg/ml streptomycin (all Invitrogen) and 2 mM L-glutamine (Lonza).

CD11c^+^ DCs and CD11c^-^ cells were sorted using a FACS Aria (BD) based on Alexa647-conjugated anti-CD11c (1:100, 301619; BioLegend). Purity of sorted cells was over 98%.

DCs were stimulated with 10 μg/ml *E*. *coli* DNA, 5 μM ODN-2216, 5 μM ODN-2006, 5 μM ODN-2395, 5 μM control ODN, 10 ng/ml LPS or 10 μg/ml R837 (all Invitrogen) unless stated otherwise. Blocking DC-SIGN antibodies (clone AZN-D1) or control isotype clone 10E2 (20 μg/ml, both produced in house [18,41]) were added 30 min prior to DC stimulation. *E*. *coli* DNA was treated for 30 min with DNAse (Promega) prior to stimulation.

RNA interference was performed by transfecting cells with 500 nM short interfering RNAs (siRNAs) using the Neon® Transfection System (ThermoFisher) according to the manufacturer’s instructions. In brief, cells were washed with PBS, resuspended in Buffer R (ThermoFisher) and transfected with a single pulse of 1500V for 20 ms. Cells were mixed with complete RMPI and incubated for 48h before stimulation. SMARTpool siRNA used were TLR7 (M-004714-01), TLR9 (M-004066-01-0005), IRF7 (M-011810-02) and non-targeting siRNA (D-001206-13) as control (all Thermo Fisher). Silencing was confirmed by real-time PCR or flow cytometry (Fig 4C and 4D; S3 Fig). Antibodies used for flow cytometry were FITC-conjugated anti-DC-SIGN (1:25, FAB161F-100, R&D Systems) and FITC-conjugated IgG1 as isotype control (1:25, 11-4714-42, Thermo Fisher). Cells were analyzed on a Canto II (BD Biosciences).

### DC maturation

DC maturation was analyzed 24 hours post stimulation. Cells were stained with PE-conjugated anti-CD80 (1:25, 557227, BD Pharmingen), APC-conjugated anti-CD83 (1:25, 551073, BD Pharmingen) and FITC-conjugated anti-CD86 (1:25; 555657; BD Pharmingen). Cells were analyzed on a FACS Canto II (BD Biosciences).

### TLR9 expression

TLR9 expression was analyzed by immunoblot. Whole-cell extracts were prepared using RIPA buffer (Cell Signaling). Proteins were resolved by SDS–polyacrylamide gel electrophoresis and detected by immunoblotting with anti-TLR9 (1:1,000; 2254; Cell Signaling) or β-actin (1:2500, sc-81178, Santa Cruz), followed by incubation with HRP-conjugated secondary antibody (1:2,500; 21230; Pierce) and ECL detection (Pierce).

TLR9 expression was also examined by flow cytometry. Cells were stained with anti-TLR9 (1:200; 2254; Cell Signaling), followed by AlexaFluor 647-conjugated anti-rabbit (1:400, A-21245, ThermoFisher) in combination with PE-conjugated anti-CD11c (1:25; 1P-529-T100; Exbio antibodies) and PerCP-conjugated anti-CD19 (1:12.5; 332780, BD Biosciences). Cells were analyzed on a FACS Canto II (BD Biosciences).

### Cellular binding

DCs or Raji cells stably expressing DC-SIGN or Langerin [41] were incubated in the presence or absence of EGTA (10 mM) or α-DC-SIGN (20 μg/ml, clone AZN-D1) for 30 min followed by ODN-2216-FITC (Invivogen) or EC-DNA-FITC (FastTag Basic Nucleic Acid Labeling Kit, Vector Laboratories) for 10 min at 37°C. Dead cells were excluded using LIVE/DEAD™ Fixable Red Dead Cell Stain (Thermo Fisher). Cells were analyzed on a FACS Calibur (BD).

### Confocal

DCs were stimulated with 5 μM ODN-2216-FITC (Invivogen) or 10 μg/ml *E*. *coli* DNA-FITC (FastTag Basic Nucleic Acid Labeling Kit, Vector Laboratories) for 20 min and allowed to adhere on poly-L-leucine-coated glass slides for 10 min at 37°C. Cells were fixed in 2% paraformaldehyde for 10 min and permeabilized in methanol for 10 min. Cells were stained with α-DC-SIGN (5 μg/ml, clone AZN-D1) and α-EEA1 (5 μg/ml, Abcam) followed with α-rabbit-Alexa546 and α-mouse-Alexa647 (1:400, both Invitrogen) and nuclei were stained with Hoechst (1:10.000, Molecular Probes). Cells were analyzed on a Leica TCS SP8 X mounted on a Leica DMI6000 inverted microscope and data was processed using Leica LAS-X software.

### ELISA

DC-SIGN-Fc was produced as previous described [31]. Synthetic ODNs or *E*. *coli* DNA was coated on immunosorbent plates for 24h at 4°C. Plates were blocked with 2% BSA in TSM (20 mM Tris-HCL, Na,150 mM Na Cl, 1 mM CaCl_2_, 2 mM MgCl_2_) for 30 min. DC-SIGN-Fc containing supernatant pretreated or not with EGTA (10 mM) or Mannan (1 mg/ml) for 30 min was added 1:1 with TSM for 2h at room temperature followed by α-Human-Fc-HRP. Optical density was measured at 450 nm. Conversely, DC-SIGN-Fc was coated on immosorbent plates and binding to biotinylated *E*. *coli* DNA (FastTag Basic Nucleic Acid Labeling Kit, Vector Laboratories) or biotinylated Fucose (Lectinity) was measured using streptavidin-HRP. *E*. *coli* DNA was treated with DNAse (Promega) for 30 min were indicated.

### Real-time quantitative PCR

mRNA was isolated using mRNA capture kit (Roche). cDNA was synthesized with reverse transcriptase kit (Promega) and PCR amplification was performed in the presence of SYBR Green in an ABI 7500 Fast PCR detection system (Applied Biosystems). Specific primers were designed using Primer Express 2.0 (Applied Biosystems; S1 Table). Expression of target genes was normalized to GAPDH (*N*_t_ = 2^Ct(GAPDH)–Ct(target)^) and set at 1 in DENV-infected DCs for each donor within one experiment.

### Statistical analysis

Statistical analyses were performed using the Student’s *t-*test for paired observations. Statistical significance was set at *P*<0.05.

## Supporting information

S1 Fig E. coli DNA does not trigger TLR2 or TLR4. Parental HEK293 cells or HEK293 cells stably expressing human TLR2 or TLR4 were stimulated with TLR2 ligand PAM3CSK4, TLR4 ligand LPS or EC-DNA for 24h. Cell culture supernatant was analyzed for IL-8 using ELISA. Data are representative of three idependent experiments (mean ± s.d. of duplicate measurements). EC-DNA: E. coli DNA.
(TIF)

S2 Fig Gating strategy of Raji, Raji-DC-SIGN, Raji-Langerin and DCs. Cells were selected on FSC-A and SSC-A and live single cells were selected using LIVE/DEAD™ Fixable Red Dead Cell Stain and FSC-W and FSC-H, respectively. Data are representative for at least four experiments with different donors.
(TIF)

S3 Fig Silencing efficiency of TLR7, TLR9 and IRF7. Silencing of indicated proteins using RNA interference was confirmed by real-time PCR. mRNA expression was normalized to GAPDH and set at 1 in cells treated with control siRNA. Data are collated (mean ± s.d.) of four (TLR7, TLR9) or two (IRF7) independent experiments with different donors. **P<0.01 (student’s t-test).
(TIF)

S4 Fig DC-SIGN expression is confined to CD11c^+^ DCs. Single cells were divided in CD11c^+^ cells and CD11c- cells and the expression of DC-SIGN was analyzed by flow cytometry. Numbers adjacent to gates indicate percentage of gated cells. Data are representative of four independent experiments with different donors.
(TIF)

S1 Table Sequences of RT-qPCR primers used. (TIF)

## References

[1] Geijtenbeek TBH, Gringhuis SI. C-type lectin receptors in the control of T helper cell differentiation. Nat Rev Immunol. 2016;16: 433–48. doi: 10.1038/nri.2016.55 27291962 10.1038/nri.2016.55

[2] Geijtenbeek TBH, Gringhuis SI. Signalling through C-type lectin receptors: shaping immune responses. Nat Rev Immunol. Nature Publishing Group; 2009;9: 465–79. doi: 10.1038/nri2569 19521399 10.1038/nri2569 PMC7097056

[3] Pasare C, Medzhitov R. Toll-like receptors: Balancing host resistance with immune tolerance. Curr Opin Immunol. 2003;15: 677–682. doi: 10.1016/j.coi.2003.09.002 14630202 10.1016/j.coi.2003.09.002

[4] Iwasaki A, Medzhitov R. Toll-like receptor control of the adaptive immune responses. Nat Immunol. 2004;5: 987–995. doi: 10.1038/ni1112 15454922 10.1038/ni1112

[5] Krieg AM. Therapeutic potential of Toll-like receptor 9 activation. Nat Rev Drug Discov. 2006;5: 471–84. doi: 10.1038/nrd2059 16763660 10.1038/nrd2059

[6] Ewald SE, Barton GM. Nucleic acid sensing Toll-like receptors in autoimmunity. Curr Opin Immunol. 2011;23: 3–9. doi: 10.1016/j.coi.2010.11.006 21146971 10.1016/j.coi.2010.11.006 PMC3057394

[7] Krieg AM. CpG motifs in bacterial DNA and their immune effects. Annu Rev Immunol. 2002;20: 709–760. doi: 10.1146/annurev.immunol.20.100301.064842 11861616 10.1146/annurev.immunol.20.100301.064842

[8] Kerkmann M, Costa LT, Richter C, Rothenfusser S, Battiany J, Hornung V, et al Spontaneous formation of nucleic acid-based nanoparticles is responsible for high interferon-?? induction by CpG-A in plasmacytoid dendritic cells. J Biol Chem. 2005;280: 8086–8093. doi: 10.1074/jbc.M410868200 15591070 10.1074/jbc.M410868200

[9] Honda C, Ohba K, Hideyuki Y, Negishi H, Mizutani T, Takaoka A, et al Spatiotemporal regulation of MyD88 –IRF-7 signalling for robust type-I interferon induction. Nature. 2005;434: 1–6. doi: 10.1038/nature03487.1 10.1038/nature03547 15815647

[10] Bode C, Zhao G, Steinhagen F, Kinjo T, and Klinman DM. CpG DNA as a vaccine adjuvant. 2012;10: 499–511. doi: 10.1586/erv.10.174.CpG 10.1586/erv.10.174 PMC3108434 21506647

[11] McHutchison JG, Bacon BR, Gordon SC, Lawitz E, Shiffman M, Afdhal NH, et al Phase 1B, randomized, double-blind, dose-escalation trial of CPG 10101 in patients with chronic hepatitis C virus. Hepatology. 2007;46: 1341–1349. doi: 10.1002/hep.21773 17929306 10.1002/hep.21773

[12] Verthelyi D, Gursel M, Kenney RT, Lifson JD, Liu S, Mican J, et al CpG oligodeoxynucleotides protect normal and SIV-infected macaques from Leishmania infection. J Immunol. 2003;170: 4717–4723. doi: 10.4049/jimmunol.170.9.4717 12707351 10.4049/jimmunol.170.9.4717

[13] Lahoud MH, Ahmet F, Zhang J, Meuter S, Policheni AN. DEC-205 is a cell surface receptor for CpG oligonucleotides. 2012; 10.1073/pnas.1208796109/-/DCSupplemental.www.pnas.org/cgi/doi/10.1073/pnas.1208796109 10.1073/pnas.1208796109 PMC3479608 22988114

[14] Moseman AP, Moseman EA, Schworer S, Smirnova I, Volkova T, von Andrian U, et al Mannose receptor 1 mediates cellular uptake and endosomal delivery of CpG-motif containing oligodeoxynucleotides. J Immunol. 2013;191: 5615–24. doi: 10.4049/jimmunol.1301438 24184555 10.4049/jimmunol.1301438 PMC3834123

[15] Lande R, Gregorio J, Facchinetti V, Chatterjee B, Wang Y-H, Homey B, et al Plasmacytoid dendritic cells sense self-DNA coupled with antimicrobial peptide. Nature. 2007;449: 564–9. doi: 10.1038/nature06116 17873860 10.1038/nature06116

[16] Sandgren S, Wittrup A, Cheng F, Jönsson M, Eklund E, Busch S, et al The Human Antimicrobial Peptide LL-37 Transfers Extracellular DNA Plasmid to the Nuclear Compartment of Mammalian Cells via Lipid Rafts and Proteoglycan-dependent Endocytosis. J Biol Chem. 2004;279: 17951–17956. doi: 10.1074/jbc.M311440200 14963039 10.1074/jbc.M311440200

[17] Engering A, Geijtenbeek TBH, van Vliet SJ, Wijers M, van Liempt E, Demaurex N, et al The Dendritic Cell-Specific Adhesion Receptor DC-SIGN Internalizes Antigen for Presentation to T Cells. J Immunol. 2002;168: 2118–2126. doi: 10.4049/jimmunol.168.5.2118 11859097 10.4049/jimmunol.168.5.2118

[18] Geijtenbeek TBH, Torensma R, van Vliet SJ, van Duijnhoven GC, Adema GJ, van Kooyk Y, et al Identification of DC-SIGN, a novel dendritic cell-specific ICAM-3 receptor that supports primary immune responses. Cell. 2000;100: 575–85. Available: http://www.ncbi.nlm.nih.gov/pubmed/10721994 10721994 10.1016/s0092-8674(00)80693-5

[19] Gringhuis SI, den Dunnen J, Litjens M, van der Vlist M, Geijtenbeek TBH. Carbohydrate-specific signaling through the DC-SIGN signalosome tailors immunity to Mycobacterium tuberculosis, HIV-1 and Helicobacter pylori. Nat Immunol. Nature Publishing Group; 2009;10: 1081–8. doi: 10.1038/ni.1778 19718030 10.1038/ni.1778

[20] Hovius JWR, De Jong M a WP, Dunnen J Den, Litjens M, Fikrig E, Van Der Poll T, et al Salp15 binding to DC-SIGN inhibits cytokine expression by impairing both nucleosome remodeling and mRNA stabilization. PLoS Pathog. 2008;4 doi: 10.1371/journal.ppat.0040031 18282094 10.1371/journal.ppat.0040031 PMC2242833

[21] van Liempt E, Bank CMC, Mehta P, García-Vallejo JJ, Kawar ZS, Geyer R, et al Specificity of DC-SIGN for mannose- and fucose-containing glycans. FEBS Lett. 2006;580: 6123–6131. doi: 10.1016/j.febslet.2006.10.009 17055489 10.1016/j.febslet.2006.10.009

[22] Geijtenbeek TBH, Kwon DS, Torensma R, Vliet SJ Van, Duijnhoven GCF Van, Middel J, et al DC-SIGN, a Dendritic Cell–Specific HIV-1-Binding Protein that Enhances trans -Infection of T Cells. Cell. 2000;100: 587–597. 10721995 10.1016/s0092-8674(00)80694-7

[23] Pisetsky DS. The origin and properties of extracellular DNA: From PAMP to DAMP. Clin Immunol. Elsevier Inc.; 2012;144: 32–40. doi: 10.1016/j.clim.2012.04.006 22659033 10.1016/j.clim.2012.04.006 PMC3724456

[24] Wang BX, Fish EN. The yin and yang of viruses and interferons. Trends Immunol. Elsevier Ltd; 2012;33: 190–197. doi: 10.1016/j.it.2012.01.004 22321608 10.1016/j.it.2012.01.004 PMC7106503

[25] Gonzalez SF, Lukacs-Kornek V, Kuligowski MP, Pitcher L a, Degn SE, Kim Y-A, et al Capture of influenza by medullary dendritic cells via SIGN-R1 is essential for humoral immunity in draining lymph nodes. Nat Immunol. Nature Publishing Group; 2010;11: 427–434. doi: 10.1038/ni.1856 20305659 10.1038/ni.1856 PMC3424101

[26] Wykes M, Pombo A, Jenkins C, MacPherson GG. Dendritic cells interact directly with naive B lymphocytes to transfer antigen and initiate class switching in a primary T-dependent response. J Immunol. 1998;161: 1313–9. 9686593

[27] Hemmi H, Takeuchi O, Kawai T, Kaisho T, Sato S, Sanjo H, et al A Toll-like receptor recognizes bacterial DNA. Nature. 2000;408: 740–745. doi: 10.1038/35047123 11130078 10.1038/35047123

[28] Krieg AM. CpG motifs in bacterial DNA and their immune effects. Annu Rev Immunol. 2002;20: 709–760. doi: 10.1146/annurev.immunol.20.100301.064842 11861616 10.1146/annurev.immunol.20.100301.064842

[29] Iwasaki A, Medzhitov R. Toll-like receptor control of the adaptive immune responses. Nat Immunol. 2004;5: 987–995. doi: 10.1038/ni1112 15454922 10.1038/ni1112

[30] Frisont N, Taylor ME, Soilleux E, Bousser MT, Mayer R, Monsigny M, et al Oligolysine-based oligosaccharide clusters: Selective recognition and endocytosis by the mannose receptor and dendritic cell-specific intercellular adhesion molecule 3 (ICAM-3)-grabbing nonintegrin. J Biol Chem. 2003;278: 23922–23929. doi: 10.1074/jbc.M302483200 12695508 10.1074/jbc.M302483200

[31] Geijtenbeek TBH, Van Duijnhoven GCF, Van Vliet SJ, Krieger E, Vriend G, Figdor CG, et al Identification of different binding sites in the dendritic cell-specific receptor DC-SIGN for intercellular adhesion molecule 3 and HIV-1. J Biol Chem. 2002;277: 11314–11320. doi: 10.1074/jbc.M111532200 11799126 10.1074/jbc.M111532200

[32] Ana M. A, Eicke L, Betty M, Sean R. C, Mark J. S, Frances L, et al Differential Cytokine Production and Bystander Activation of Autoreactive B Cells in Response to CpG-A and CpG-B ODNs1. J Immunol. 2009;76: 211–220. doi: 10.1007/s11103-011-9767-z.Plastid 10.4049/jimmunol.0901941 PMC3426913 19864612

[33] Gehrke N, Mertens C, Zillinger T, Wenzel J, Bald T, Zahn S, et al Oxidative damage of dna confers resistance to cytosolic nuclease trex1 degradation and potentiates STING-dependent immune sensing. Immunity. 2013;39: 482–495. doi: 10.1016/j.immuni.2013.08.004 23993650 10.1016/j.immuni.2013.08.004

[34] Geijtenbeek TBH, Van Vliet SJ, Koppel E a, Sanchez-Hernandez M, Vandenbroucke-Grauls CMJE, Appelmelk B, et al Mycobacteria target DC-SIGN to suppress dendritic cell function. J Exp Med. 2003;197: 7–17. doi: 10.1084/jem.20021229 12515809 10.1084/jem.20021229 PMC2193797

[35] Takahara K, Yashima Y, Omatsu Y, Yoshida H, Kimura Y, Kang YS, et al Functional comparison of the mouse DC-SIGN, SIGNR1, SIGNR3 and Langerin, C-type lectins. Int Immunol. 2004;16: 819–829. doi: 10.1093/intimm/dxh084 15096474 10.1093/intimm/dxh084

[36] Costerton JW, Stewart PS, Greenberg EP, Lawrence JR, Korber DR, Hyde BD, et al Bacterial biofilms: a common cause of persistent infections. Science. 1999;284: 1318–22. doi: 10.1126/science.284.5418.1318 10334980 10.1126/science.284.5418.1318

[37] Liu HH, Yang YR, Shen XC, Zhang ZL, Shen P, Xie ZX. Role of DNA in bacterial aggregation. Curr Microbiol. 2008;57: 139–144. doi: 10.1007/s00284-008-9166-0 18491189 10.1007/s00284-008-9166-0

[38] Das T, Sharma PK, Busscher HJ, Van Der Mei HC, Krom BP. Role of extracellular DNA in initial bacterial adhesion and surface aggregation. Appl Environ Microbiol. 2010;76: 3405–3408. doi: 10.1128/AEM.03119-09 20363802 10.1128/AEM.03119-09 PMC2869138

[39] Goodman SD, Obergfell KP, Jurcisek J a, Novotny L a, Downey JS, Ayala E a, et al Biofilms can be dispersed by focusing the immune system on a common family of bacterial nucleoid-associated proteins. Mucosal Immunol. 2011;4: 625–637. doi: 10.1038/mi.2011.27 21716265 10.1038/mi.2011.27

[40] Kostakioti M, Hadjifrangiskou M, Hultgren SJ. Bacterial biofilms: development, dispersal, and therapeutic strategies in the dawn of the postantibiotic era. Cold Spring Harb Perspect Med. 2013;3: 1–23. doi: 10.1101/cshperspect.a010306 23545571 10.1101/cshperspect.a010306 PMC3683961

[41] de Witte L, Nabatov A, Pion M, Fluitsma D, de Jong M a WP, de Gruijl T, et al Langerin is a natural barrier to HIV-1 transmission by Langerhans cells. Nat Med. 2007;13: 367–71. doi: 10.1038/nm1541 17334373 10.1038/nm1541

