# Supplementary figures and images for: DCs facilitate B cell responses against microbial DNA via DC-SIGN
Source: PLoS One. 2017 Oct 4;12(10):e0185580. doi: 10.1371/journal.pone.0185580 (PMC5627929; doi:10.1371/journal.pone.0185580)

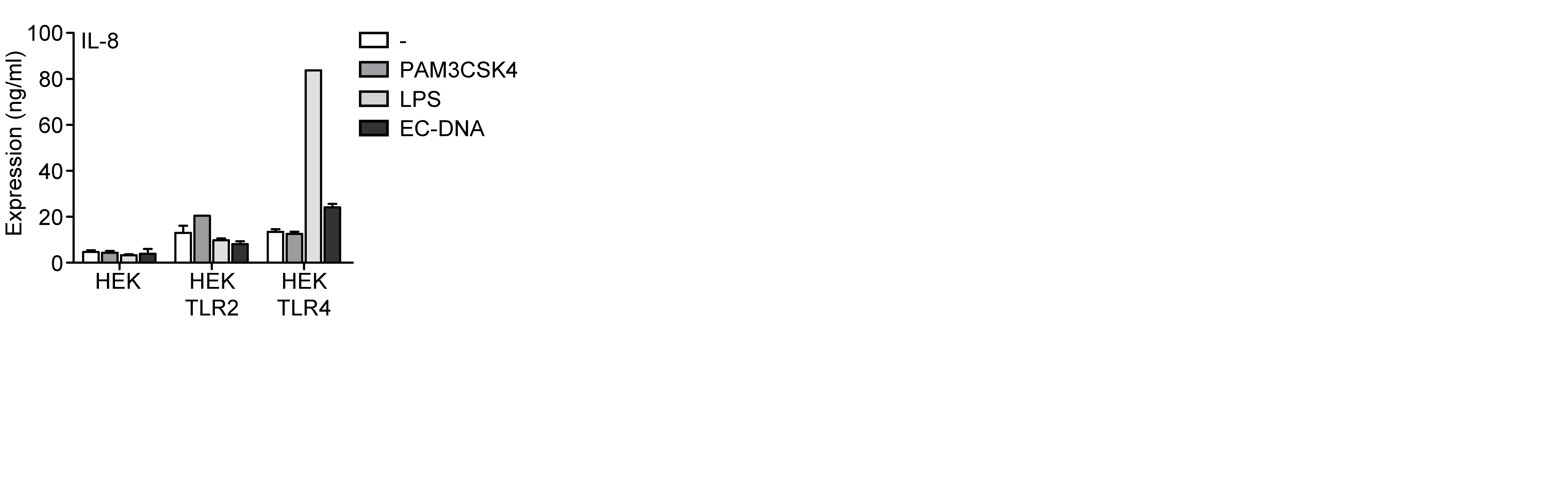

Supplement: S1 Fig — Parental HEK293 cells or HEK293 cells stably expressing human TLR2 or TLR4 were stimulated with TLR2 ligand PAM3CSK4, TLR4 ligand LPS or EC-DNA for 24h. Cell culture supernatant was analyzed for IL-8 using ELISA. Data are representative of three idependent experiments (mean ± s.d. of duplicate measurements). EC-DNA: E. coli DNA. (TIF) [file pone.0185580.s001.tif]

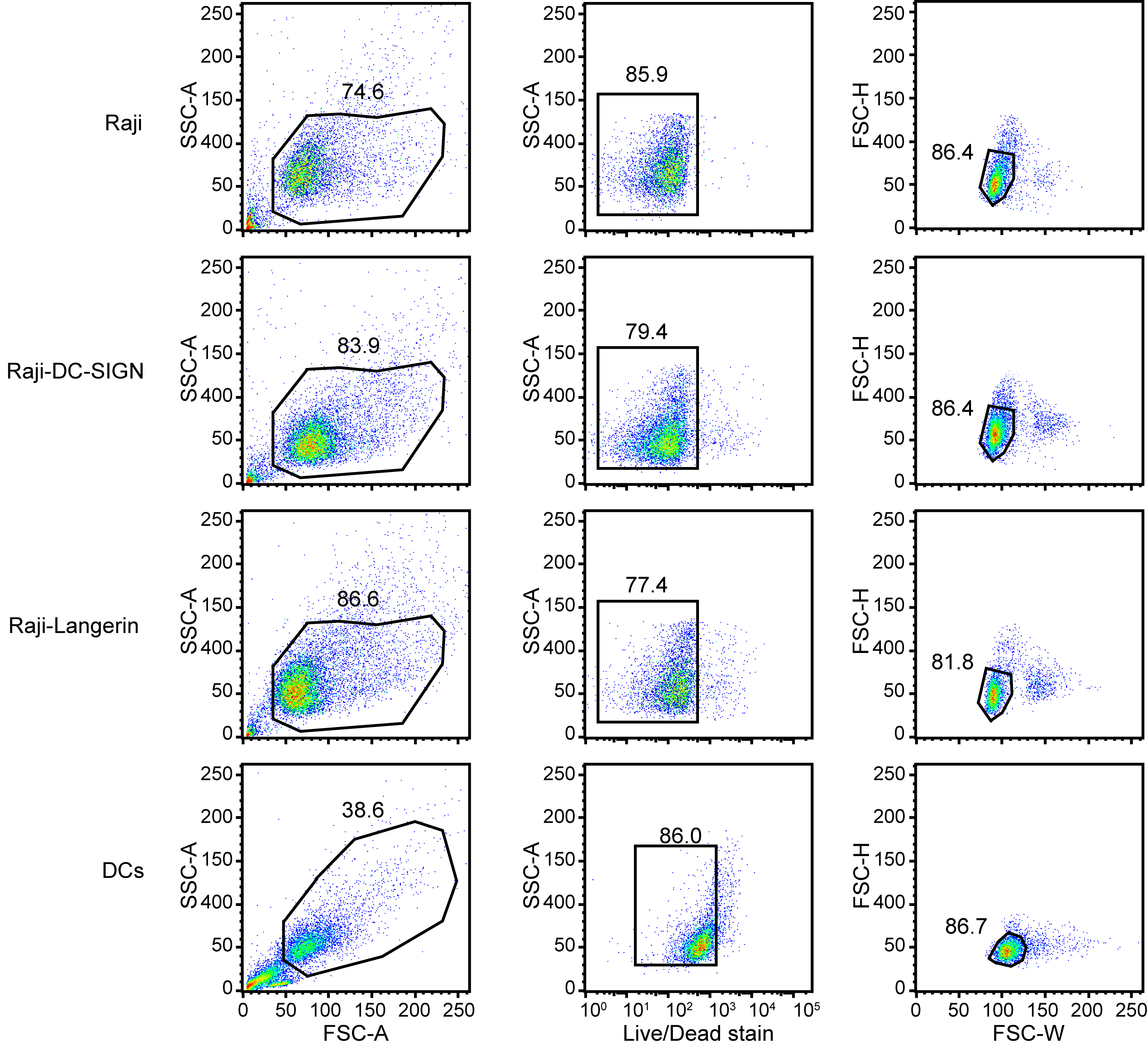

Supplement: S2 Fig — Cells were selected on FSC-A and SSC-A and live single cells were selected using LIVE/DEAD™ Fixable Red Dead Cell Stain and FSC-W and FSC-H, respectively. Data are representative for at least four experiments with different donors. (TIF) [file pone.0185580.s002.tif]

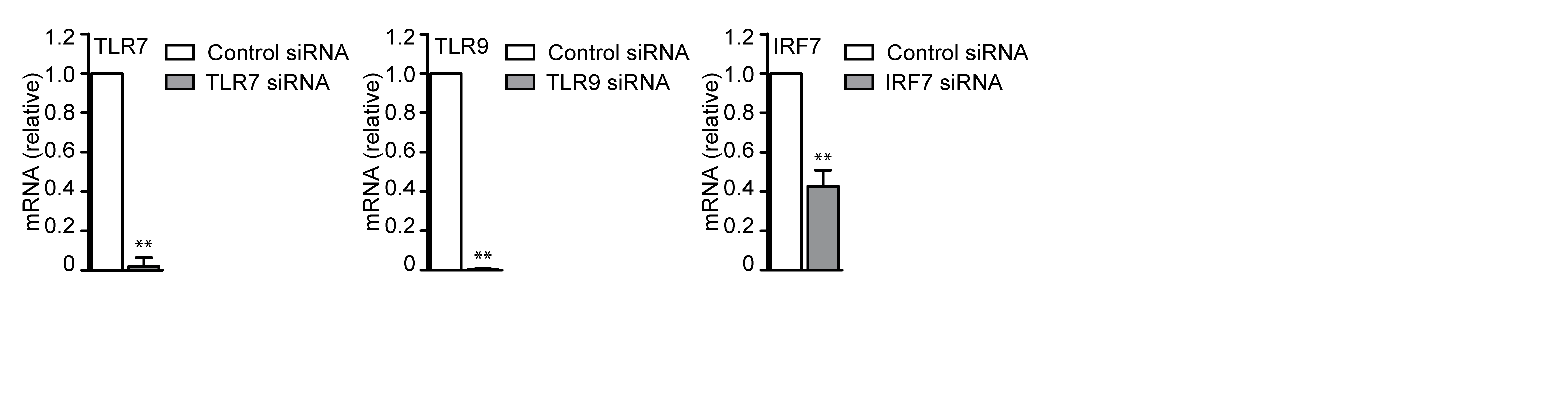

Supplement: S3 Fig — Silencing of indicated proteins using RNA interference was confirmed by real-time PCR. mRNA expression was normalized to GAPDH and set at 1 in cells treated with control siRNA. Data are collated (mean ± s.d.) of four (TLR7, TLR9) or two (IRF7) independent experiments with different donors. **P<0.01 (student’s t-test). (TIF) [file pone.0185580.s003.tif]

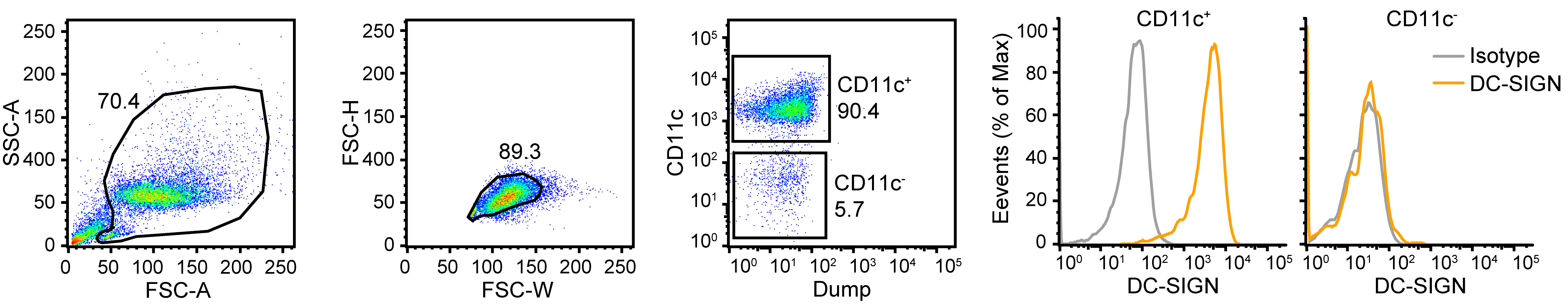

Supplement: S4 Fig — Single cells were divided in CD11c+ cells and CD11c- cells and the expression of DC-SIGN was analyzed by flow cytometry. Numbers adjacent to gates indicate percentage of gated cells. Data are representative of four independent experiments with different donors. (TIF) [file pone.0185580.s004.tif]

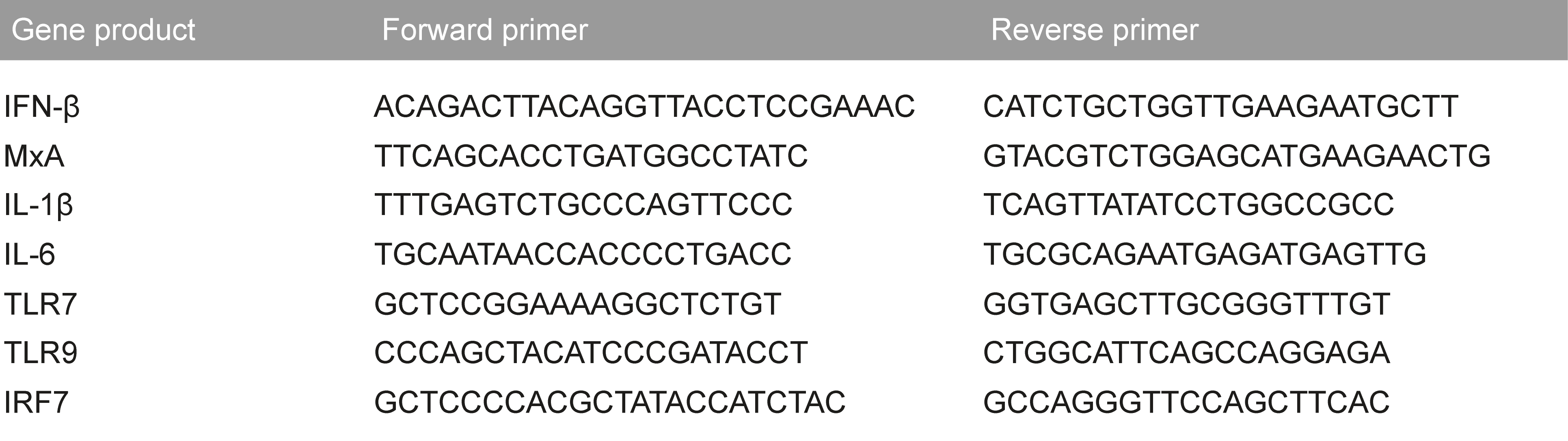

Supplement: S1 Table — (TIF) [file pone.0185580.s005.tif]
